# Bariatric Surgery for Type 2 Diabetes Mellitus in Patients with BMI <30 kg/m^2^: A Systematic Review and Meta-Analysis

**DOI:** 10.1371/journal.pone.0132335

**Published:** 2015-07-13

**Authors:** Attit Baskota, Sheyu Li, Niharika Dhakal, Guanjian Liu, Haoming Tian

**Affiliations:** 1 Department of Endocrinology and Metabolism, West China Hospital, Sichuan University, Chengdu, China; 2 Department of Anesthesiology and Pain management, West China Hospital, Sichuan University, Chengdu, China; 3 Chinese Evidence-Based Medicine/Cochrane Center, Chengdu, China; University of Texas Health Science Center at San Antonio, UNITED STATES

## Abstract

**Background and Objective:**

The role of bariatric surgery in non-obese patients with type 2 diabetes (T2DM) remains unclear, and its use in clinical practice is controversial. We conducted a systemic review and meta-analysis to investigate the metabolic changes after surgical treatment in diabetic patients with body mass index (BMI) <30 kg/m^2^.

**Methods:**

We conducted a comprehensive search in MEDLINE (PubMed), EMBASE and the Cochrane Library of published articles from January 2000 to April 2013 reporting the clinical outcome changes in various metabolic outcomes in diabetic patients with BMI <30 kg/m^2^.

**Results:**

Ten prospective studies including 290 patients were included in the meta-analysis. Bariatric surgery led to an overall 2.79 kg/m^2^ [95%CI 2.05~3.53, P<0.00001] reduction in BMI, a 1.88%[95%CI 1.32~2.43, P<0.00001] reduction in glycosylated hemoglobin, a 3.70 mmol/L [95%CI, 1.93~5.47, P<0.00001] reduction in fasting blood glucose, a 6.69 mmol/L [95%CI, 2.29~11.08, P=0.003] reduction in postprandial glucose, anda 3.37 [95%CI 0.55~6.18, P=0.02] reduction in homeostasis model assessment of insulin resistance (HOMA-IR). After surgical treatment, 76.2% of the patients were insulin free, and 61.8% patients were off medication. In total, 90(42.4%), 10(37%) and 34(37.2%) patients had post-surgical HbA1c levels of <6%,<6.5% and<7%, respectively. No deaths were observed in the included studies, and the major complication rate was 6.2%.

**Conclusions:**

Based on the currently available data, bariatric surgery might improve glycemic control and weight loss in a very limited range with a doubled surgical complication rate in drug-refractory T2DM patients with BMI <30 kg/m^2^. It remains too premature to suggest bariatric surgery for non-obese T2DM patients.

## Introduction

Type 2 diabetes mellitus (T2DM) is a global public health issue. The global prevalence of diabetes among adults 20~79 years of age was 8.3% in 2011, with 366 million people affected, and was predicted to increase to 9.9% by 2030 [1]. Current treatments for T2DM focus on the prevention and management of complications, instead of on a radical cure, because T2DM is considered to be an irreversible, chronic, and progressive disease. In spite of the rapid progress in pharmacological and non-pharmacological approaches to diabetes in recent years, 92.7% of adult diabetic patients had poorly-controlled blood glucose levels and related co-morbidities [2].

Bariatric surgery in patients with a larger body-mass index (BMI) could lead to complete resolution of diabetes in more than 90% of patients, with a reduction in risk factors for cardiac disease including hypertension and lipid abnormalities [3,4]. The underlying mechanisms have been suggested to involve weight loss and caloric restriction. However, a large study including 608 patients with a follow-up period of 14 years suggested that the resolution of diabetes occurred long before significant weight loss, and the correction of T2DM continued even when the patients remained obese [5].

In recent years, surgical treatments have attracted growing interest as therapies for non-obese T2DM patients [6–9]. Surgery has been reported to possibly facilitate glycemic control with a reduction in the need for insulin [10]. However, a limited study population and short follow-up duration might introduce biases into the outcomes. There have been few published systematic reviews on the effect of metabolic surgery as a single treatment in non-obese T2DM patients.

In this study, we attempted to analyze the published surgical observational studies in non-obese T2DM patients (defined as patients with BMI <30 kg/m^2^) to discover the clinical evidence of metabolic surgery for the resolution of diabetes.

## Materials and Methods

The meta-analysis was conducted following the Meta-analysis of Observational Studies in Epidemiology (MOOSE) guidelines [11]. We reported this study in accordance with the preferred reporting items for systematic reviews and the meta-analysis (PRISMA) checklist (S1 Table).

### Literature Search

We conducted a comprehensive search in MEDLINE (PubMed), EMBASE and the Cochrane Library from January 2000 to April 2013 with a combination of the following keywords: “bariatric surgery OR metabolic surgery OR obesity surgery OR Roux en Y OR gastric banding OR anastomosis OR biliopancreatic diversion OR gastric bypass OR jejunoileal bypass” AND “diabetes OR diabetes mellitus OR type 2 diabetes OR T2DM” AND “overweight OR low BMI OR body mass index <30 kg m2 OR normal weight”. In addition, we reviewed the references of the included studies for additional potentially eligible studies. We checked the studies for duplicate publications. Articles on identical participants were considered to use an overlapping population, under which circumstances, the data were extracted only from the highest quality study. Articles that only provided abstracts were included if sufficient data were reported; unpublished reports were not considered.

### Inclusion and Exclusion Criteria

The following criteria were used for inclusion of the study into the analysis:1) T2DM patients with baseline BMIs lower than 30 kg/m^2^; 2) gastrointestinal (GI) abnormalities, such as peptic ulcer diseases (PUDs), gynecological abnormalities and gastric carcinoma, were not associated at baseline; 3) at least two of the outcomes of interest were reported clearly; and 4) English was the language of publication regardless of the research methods.

Literature reviews were excluded as were articles with data from overlapping populations, animal or in vitro studies, studies involving children or adolescents, and studies of type 1 diabetes or gestational diabetes.

### Data extraction and quality assessment

All the data were extracted independently by two authors (AB, SL) using a predefined standardized data extraction form. Discrepancies were resolved by consulting a third investigator (GL). Corresponding or first authors were contacted through E-mail in cases in which the data regarding our outcome of interest were likely to have been analyzed, although they were not clearly reported. The following data were extracted from the included articles: the first author, publication year, country, study design, sample size, intervention type, sex, age, follow-up duration, diabetes duration and comparable outcomes. The following outcomes were analyzed to assess the metabolic status before and after various bariatric surgeries: the BMI, glycated hemoglobin A1c (HbA1c), fasting blood glucose (FBG), postprandial blood glucose (PP), C-peptide, homeostatic model of insulin resistance (HOMA-IR), total cholesterol (TC), triglyceride (TG) levels and body weight.

The quality of the included studies was assessed by the Newcastle-Ottawa scale for the selection of the participants, adjustments of the confounders, description of the outcomes and duration of the follow-up [12].

### Data analysis

The following data were collected to indicate diabetes-related clinical outcomes: the remission rate (the percentage of patients who had reached various target points) in each study and the medicine-free rate (the percentage of patients who discontinued medicine after surgery). The overall remission rates were calculated as the percentage of the patients having achieved an HbA1c level <6%, <6.5%, and <7%, respectively, if reported in the studies. The status of the patients receiving insulin and post-surgical amelioration of medication treatment were assessed if provided in the studies.

The status of co-morbidities and complications before surgery and the improvement rates after surgery were described based on the information provided in each study and were calculated in percentages when the relevant data were available.

The safety of the surgical methods was evaluated in percentages by the surgery-related complications and mortality rate in each included study.

### Statistical analysis

The statistical analysis was performed using Review manager 5.1 software (Copenhagen: The Nordic Cochrane Centre, The Cochrane Collaboration). A P value <0.05 was considered to be statistically significant. The mean difference (MD) and 95% CI were used to describe the continuous data for each study. We assessed the heterogeneity among the studies initially by graphically examining the forest plots and subsequently by a statistical evaluation using a Chi-square test of homogeneity and evaluation of the inconsistency index (I^2^) statistic. A P-value <0.1 or I^2^>50% indicates significant statistical heterogeneity among the studies. We pooled the studies using a random-effect model in the presence of statistical or other heterogeneity and fixed-effect models otherwise.To explore the sources of between study heterogeneity in the pooled analysis, subgroup analyses were performed based on the geographic area, intervention type, duration of diabetes, and follow-up period.

## Results

### Search results

**Fig 1:**
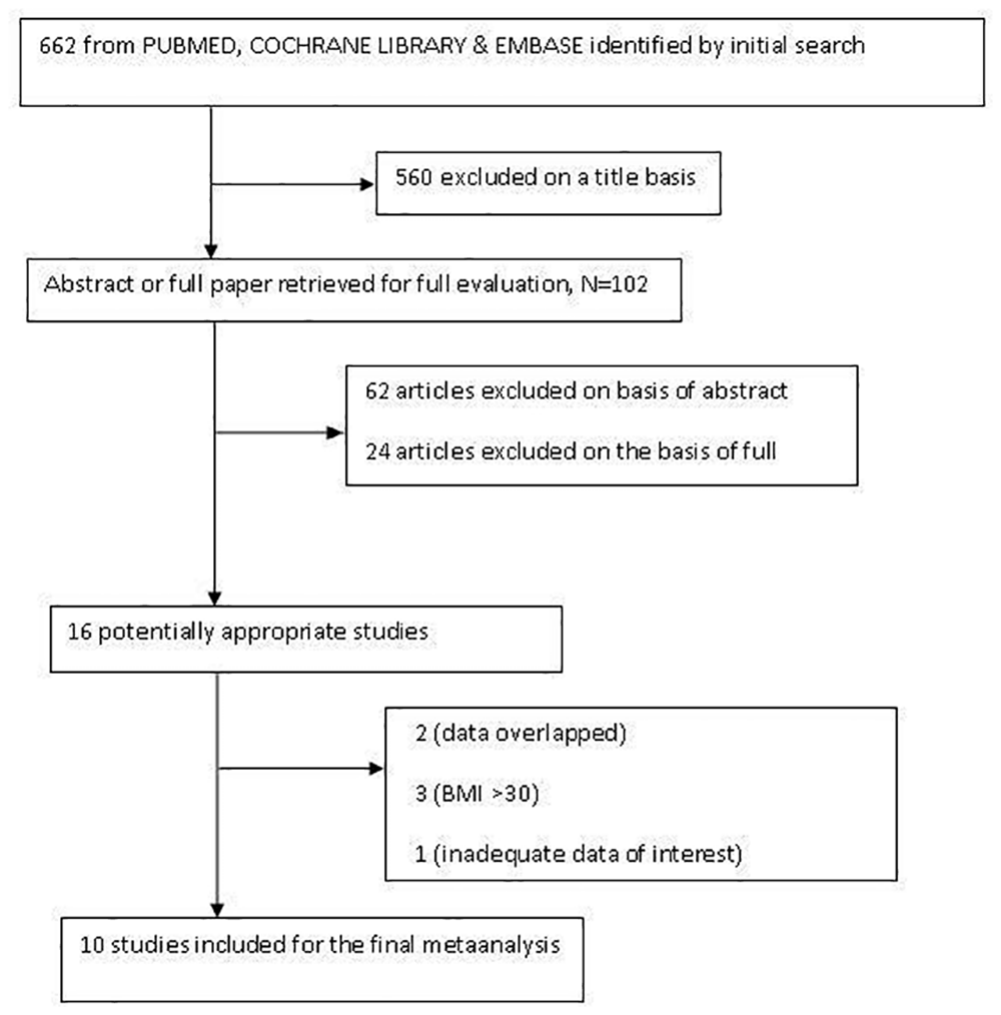
Flow chart of article selection.

The flow diagram of the article selection is shown in Fig 1. In total, 662 articles and abstracts were identified by the initial searches, of which 560 articles were excluded by screening the titles. An additional 62 papers were excluded after reading the abstracts, leaving 64 articles for the full publication review. After a full-text examination, 16 potentially appropriate papers were finally retrieved. We found population overlapping between two studies. Of two studies conducted by Depaula et al., only one reported our outcomes of interest, and it was included. Three studies had data with a BMI >30 kg/m^2^, and one study had inadequate data of interest. Ten articles were included in the final analysis [7–10,13–18]. Table 1 shows the baseline characteristics of the final studies that were included for the meta-analysis. The quality assessments of the included studies are shown in S2 Table.

**Table 1:** Baseline characteristics of the included studies.

Included study	Study design	Sample size (M/F)	Type of surgery	Country	Mean age(yrs)	Follow-up period(months)	Duration of diabetes (yrs)	Studied outcomes
Ramos et al [7]	Prospective	20 (11/9)	DJB	Brazil	43yrs	6	5.3, range: (2–8)	BMI, FBG, HbA1c, C-peptide
Depaula et al [13]	Prospective	69 (47/22)	LII+DSG	Brazil	51±5.6	21.7	11±4	FBG, HbA1c, BMI, lipids, C-peptide, HOMA-IR, co-morbidities
Geloneze et al [10]	Prospective	12 (9/3)	DJB	Brazil	50±5.3	6	9±2	FBG, HbA1c, C-peptide, BMI, C-peptide
Lee et al [18]	Prospective	6 (6/0)	DJB	Korea	50.2	6	5.5, range: (2–10)	FBG, HbA1c, body weight
Kim et al [8]	Prospective	10 (2/8)	LMGB	Korea	49.6	6	6.6	BMI. HbA1c, FBG, PP
Scopinaro et al [17]	Prospective	15 (13/2)	BPD	Italy	57.8±6.7	24	11.1±6.1	Body weight, BMI, HbA1c, HOMA-IR, lipids, FBG
Navarrete et al [9]	Prospective	10 (5/5)	LSG+DJB	Venezuela	46.5	12	<10	HbA1c, body weight, FBG, BMI
M.García et al [16]	Prospective	13 (10/3)	BAGUA	Spain	63.84±8.25	6	16.9±8.75	FBG, PP, HbA1c, C-peptide, BMI, co-morbidities.
J.B.Dixon et al [37]	Prospective	103 (41/62)	LMGB+RYGB	Korea+Taiwan	47.5±9.6	12	8.2±5.0	BMI, HbA1c
C.Shrestha et al [15]	Prospective	33 (24/9)	RYGB	China	49.51±1.33	3	<10	BMI, HbA1c, FBG, PP

BAGUA = one anastomosis gastric bypass;BMI = body mass index; BPD = biliopancreatic diversion; FBG: fasting blood glucose; DJB = duodenojejunalbypass; HOMA-IR: homeostatic model of insulin resistance; M/F = male/female; LII-DSG = laparoscopic sleeve gastrectomy; LMGB = laparoscopic mini gastric bypass;PP = postprandial blood glucose; RYGB = roux-en-Y gastric bypass; TC = total cholesterol; TG = triglyceride

### Systematic review

The ten eligible studies were prospective studies involving a total of 290 T2DM patients, with a mean age of 51.4 years, and with 58% of the pooled participants being male. The studies were conducted in populations from Brazil (3), Korea (2), Italy (1), Venezuela (1), China (1), Spain (1) and both Taiwan & Korea (1). The mean BMI was 26.62±2.19 kg/m^2^. The follow-up interval ranged from three months to two years. The mean duration of diabetes prior to surgery in each included study ranged from 2 to 20 years. The participants involved underwent bariatric surgery for the purpose of glycemic control.

Various surgical procedures were investigated, with the performance of duodenal—jejunal bypass surgery (DJB) in three studies, sleeve gastrectomy (LII-DSG) in two, biliopancreatic diversion (BPD) in one, Roux-En Y in one, laparoscopic mini-gastric bypass (LMGB) in one, and anastomosis gastric bypass (BAGUA) in one study. One study population was composed of 79% patients undergoing LMGB and 21% Roux-En Y gastric bypass. All of the patients were under treatment for diabetes, specifically by therapy with insulin, oral anti-hyperglycemic agents or both. Insulin users accounted for 42.8% of the pooled population.

Resolution and remission of T2DM was defined and reported differently in each study. In the overall population, the rates of achievement of HbA1c levels of 6%, 6.5%, and 7% were 42.4% (N = 90/212),37% (N = 10/27), and 37.2% (N = 34/94), respectively. Although the remission rate was low,the reliance on anti-diabetic medications was reduced with statistical significance after surgery. Throughout the follow-up period after surgery, 76.2% of the patients were insulin free, and 61.8% were medication free for blood glucose control. The prevalence of the co-existence of hypertension and dyslipidemia was 21.7% and 41.7% in the overall population before surgery, respectively. After surgery, blood pressure was controlled in 88.9% of the patients without antihypertensive medications, and serum TC and TG improved in 45.8% and 38% of the overall patients, respectively [13,17].

The overall major surgical complication rate was 6.2%, including intestinal obstruction, intestinal perforation, and intra-abdominal bleeding (S3 Table). The rate of early surgical complications (<30 days) was 3.4%, including the presence of a fistula, gastrointestinal bleeding, urinary tract infection, pneumonia, and wound infection. In one of the included studies with a long follow-up period (21.7 months), 15.9% of the patients reported complications including prolonged diarrhea, gouty attacks, prolonged emesis, urinary tract infection, or fungal esophagitis during the follow-up [13]. Death was not reported in any of the included studies. More detailed data from each study are provided in S4 Table.

### Meta-analyses

#### BMI and weight loss

**Fig 2:**
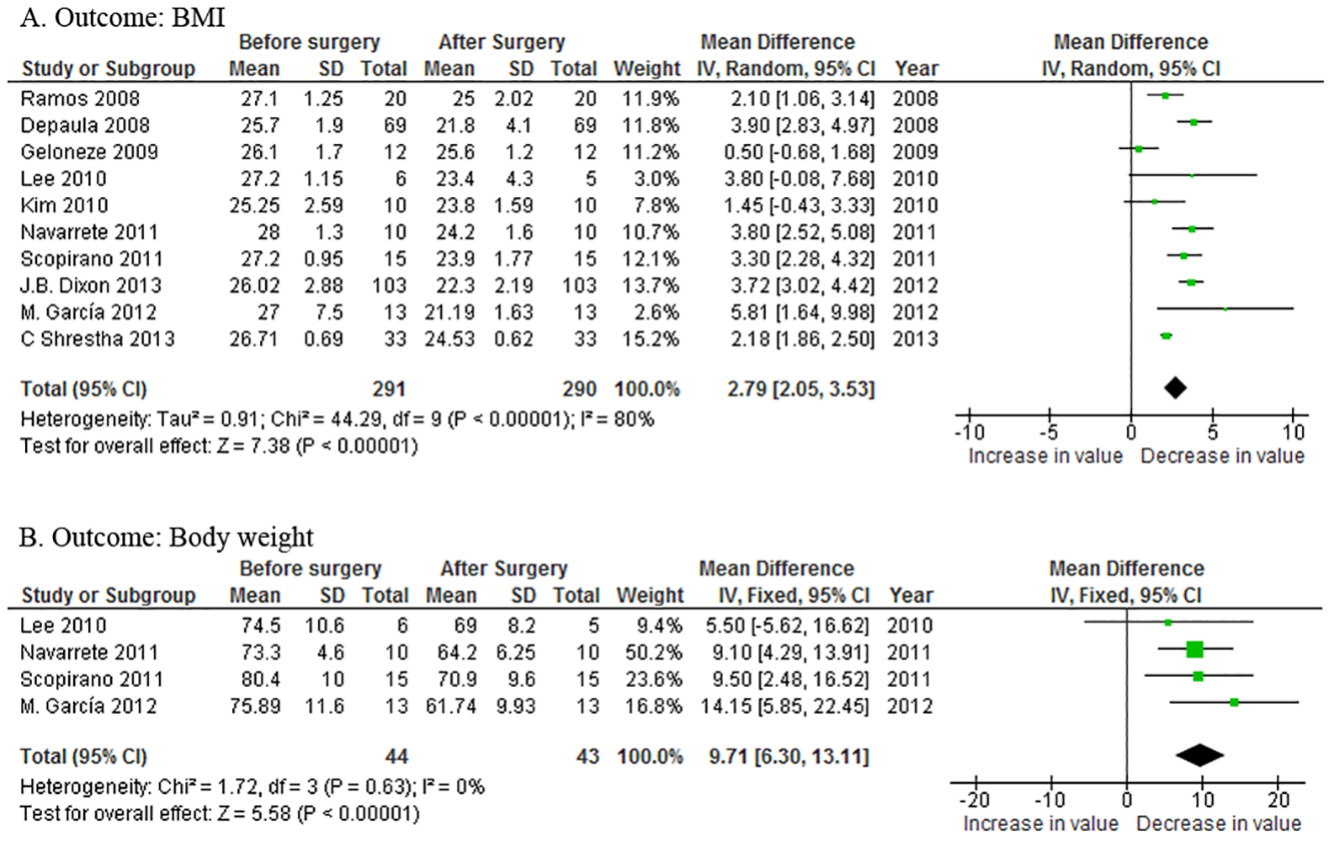
Forest plots of the effects of metabolic surgery on:(A) body mass index (BMI) and (B) body weight. CI = confidence interval; IV = inverse variance; SD = standard deviation.

All the articles reported mean changes in the BMI. Compared with the preoperative status, the BMI reduction was 2.79 kg/m^2^ [95%CI 2.05~3.53, *P*<0.00001] after surgery (Fig 2A), when a random-effect model was applied because the heterogeneity among the studies was obvious (*P*<0.0001). Body weight loss was reported in four papers, and the overall weight loss was 9.71 kg [95%CI 6.30~13.11, *P*<0.00001] (Fig 2B) in the fixed-effect model.

#### Glycemic control

**Fig 3:**
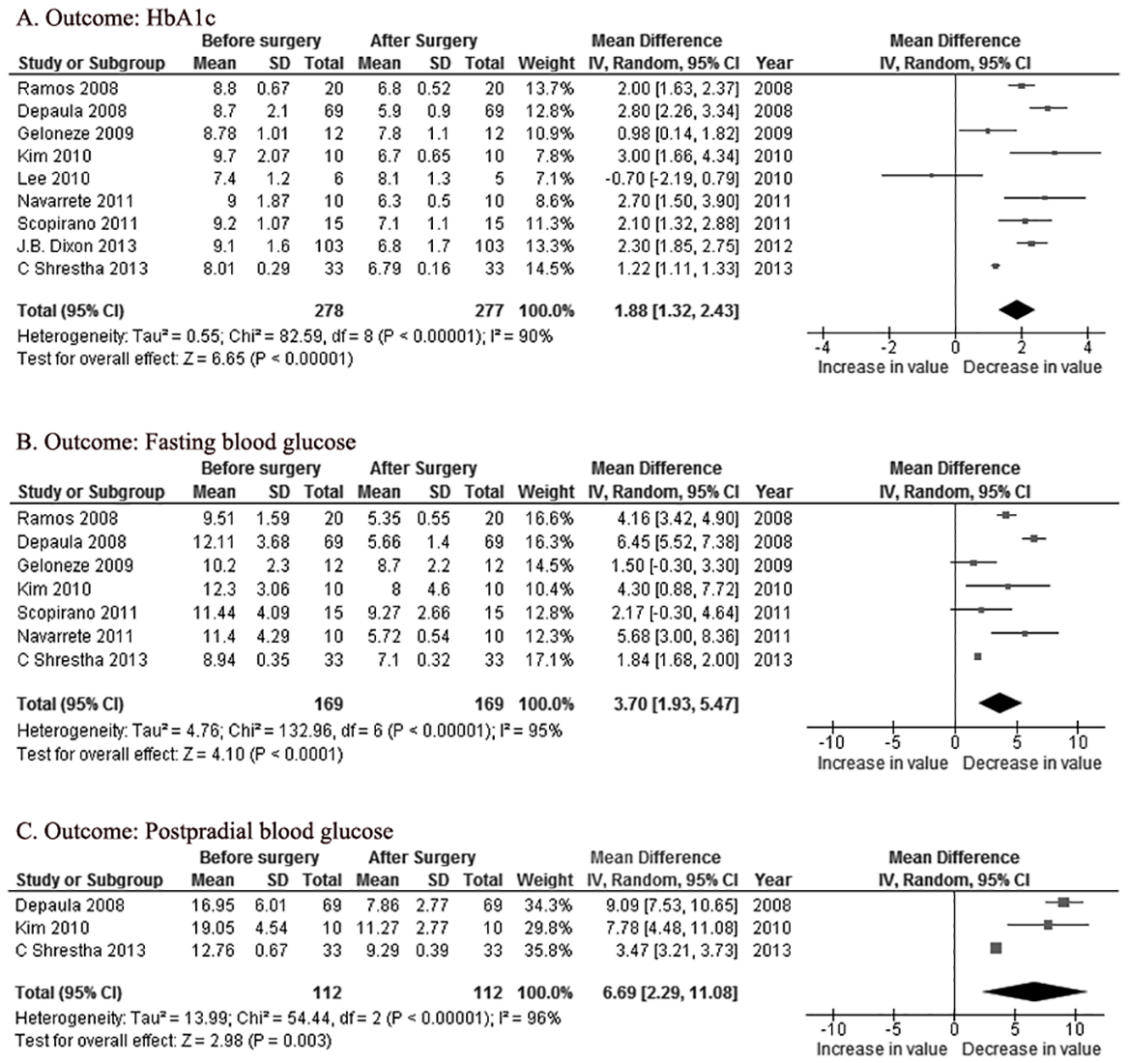
Forest plotsof the effects of metabolic surgery on: (A) HbA1c; (B) fasting blood glucose; and (C) postpradial blood glucose. CI = confidence interval; IV = inverse variance; SD = standard deviation.

Nine of the papers included in the meta-analysis reported changes in the HbA1c levels before and after surgery. The overall HbA1c level reduction was 1.88% [95%CI 1.32~2.43, *P*<0.00001] after surgery (Fig 3A). The overall reduction of the fasting blood glucose (FBG) level was 3.70mmol/L [95%CI 1.93~5.47, *P*<0.0001] in seven papers with available data (Fig 3B). The overall reduction of the postprandial blood glucose level was 6.69mmol/l [95%CI 2.29~11.08, *P* = 0.003] in three papers with available data (Fig 3C). The random—effect model was used in all three analyses because significant statistical heterogeneity among the studies was observed (*P*<0.00001 in all three analyses).

#### Lipid profiles

**Fig 4:**
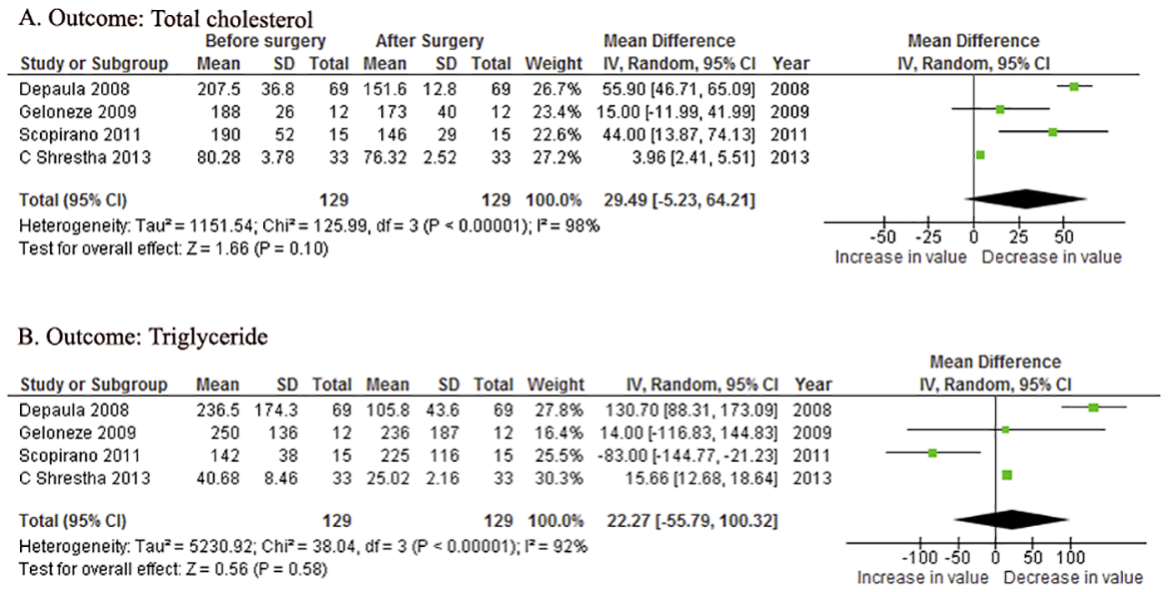
Forest plotsof the effects of metabolic surgery on: (A) total cholesterol and (B) triglyceride. CI = confidence interval; IV = inverse variance; SD = standard deviation.

Four of ten articles reported the values of TC and TG before and after surgery and were included for the mean estimation. The overall reductions of TC and TG were 29.49 mg/dl [95%CI -5.23~64.21, *P* = 0.10] and 22.27mg/dl [95%CI -55.79~100.32, *P* = 0.58], respectively (Fig 4A and 4B). The random—effect model was applied for significant statistical heterogeneity (*P*<0.0001).

#### Insulin resistance and beta cell function

**Fig 5:**
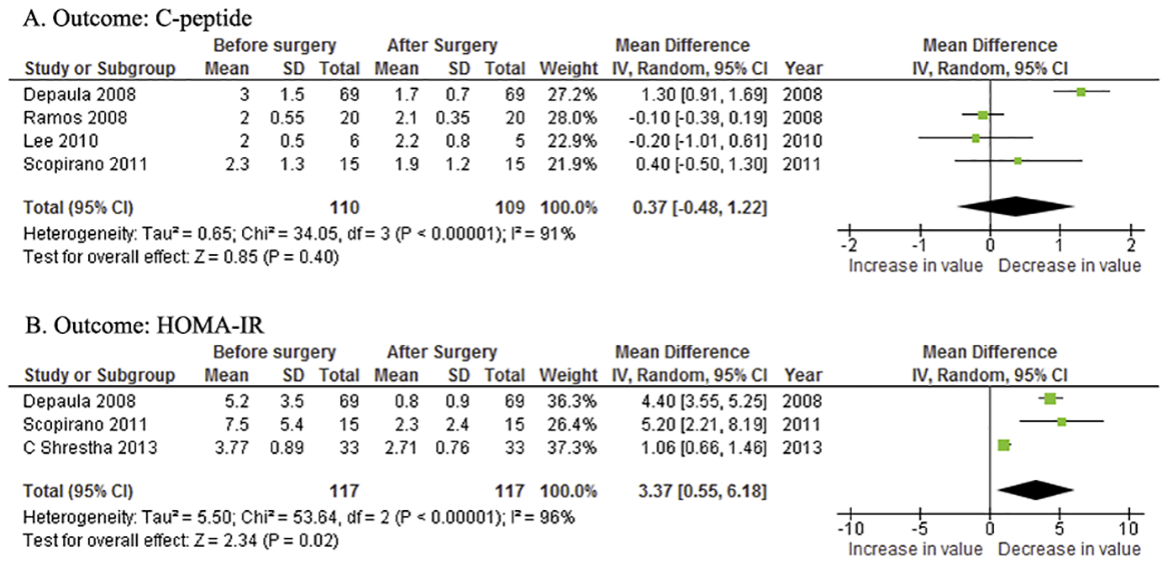
Forest plotsof the effects of metabolic surgery on: (A) C-peptide and (B) HOMA-IR. CI = confidence interval;HOMA-IR = homeostasis model assessment of insulin resistance; IV = inverse variance; SD = standard deviation.

Three articles reported the HOMA-IR before and after surgery. The overall C-peptide decrement was 0.37ng/ml [95%CI -0.48~1.22, *P* = 0.40] in four papers with reported data (Fig 5A). The overall reduction of the HOMA-IR was 3.37 [95%CI 0.55~6.18, *P* = 0.02] (Fig 5B). The random—effect model was usedin both analyses for significant statistical heterogeneity (*P*<0.00001 in both analyses).

### Subgroup analyses

We introduced subgroup analyses to explore the source of heterogeneity and understand the effect factors of the results better. We explored all the studied outcomes, stratified by the follow-up period, type of surgery, duration of diabetes, and ethnicity. The results are shown in Table 2. In the subgroup with a follow-up period of longer than seven months, the pooled BMI, HbA1c, and FBG showed a significant reduction (pooled OR [95% CI]: BMI, 3.68 [3.20~4.15]; HbA1c, 2.46 [2.15~2.76]; FBG, 4.91 [2.32~7.51]), all of which were more significant reduced than those in the subgroup with a shorter follow-up period (pooled OR [95% CI]: BMI, 2.08 [1.79~2.37]; HbA1c, 1.28 [1.18~1.39]; FBG, 2.80 [1.16~4.45]). This finding indicated that the effect of bariatric surgery might be associated with the follow-up duration. In the subgroup with a duration of diabetes longer than eight years, the pooled BMI and HbA1c levels were reduced significantly (pooled OR [95% CI]: BMI, 3.41 [2.51~4.31]; HbA1c, 2.18 [1.38~2.98]), both of which were more significantly reduced than those in the subgroup with a shorter diabetic duration (pooled OR [95% CI]: BMI, 2.00 [1.32~2.68]; HbA1c, 1.78 [1.46~2.11]). This finding indicated that patients with a shorter duration of diabetes might gain greater benefit from bariatric surgery.

**Table 2:** Stratified analyses of the investigated outcomes.

**Variables**	**Stratification**	**Number of studies**	**Pooled mean differences (95%CI)**	**P value for heterogeneity**	**I** ^**2**^	**P value for pooled mean differences**
****BMI****						
**** Follow-up period****						
	>7 months	4	3.68 [3.20–4.15]	p = 0.86	0%	p<0.0001
	<7 months	5	2.08 [1.79, 2.37]	p = 0.06	56%	p<0.0001
**** Type of surgery****						
	DJB	3	2.20 [1.29, 3.11]	p = 0.43	0%	p<0.0001
**** Duration of diabetes****						
	>8 years	6	3.41 [2.51,4.31]	p = 0.0001	83%	p<0.00001
	<8 years	4	2.00 [1.32, 2.68]	p = 0.93	0%	p<0.0001
**** Ethnics****						
	Asia	4	2.55 [1.58, 3.51]	p = 0.0009	82%	p<0.0001
	South America	4	3.08 [2.46, 3.70]	p = 0.04	65%	p<0.0001
****HbA1c****						
**** Follow-up period****						
	>7 months	4	2.46 [2.15, 2.76]	p = 0.39	0%	p<0.00001
	<7 months	5	1.28 [1.18, 1.39]	p = 0.0005	86%	p<0.0001
**** Type of surgery****						
	DJB	3	1.71 [1.38, 2.04]	p = 0.00001	87%	P = 0.16
**** Duration of diabetes****						
	>8years	4	2.18 [1.38, 2.98]	p = 0.00001	93%	p<0.00001
	<8years	4	1.78 [1.46, 2.11]	p = 0.001	84%	p = 0.01
**** Ethnics****						
	Asia	3	1.55 [0.60, 2.50]	p = 0.00001	91%	0 = 0.001
	South America	4	2.14 [1.86, 2.42]	p = 0.006	79%	p<0.0001
****FBG****						
**** Follow-up period****						
	>7 months	3	4.91 [2.32, 7.51]	p<0.00001	80%	p<0.0002
	<7 months	4	2.80[1.16, 4.45]	p = 0.007	92%	p<0.0008
**** Type of surgery****						
	DJB	2	2.96 [0.37, 5.56]	p<0.00001	86%	p = 0.03
**** Duration of diabetes****						
	>8 years	4	4.02[0.97, 7.07]	p = 0.00001	97%	p = 0.010
	<8 years	3	3.27 [1.31, 5.22]	p = 0.03	72%	p = 0.001
**** Ethnics****						
	Asia	2	2.45 [0.37, 4.53]	p = 0.02	49%	p = 0.02
	South America	4	4.46 [2.53, 6.39]	p = 0.00001	89%	p<0.0001
****PP****						
**** Follow-up period****						
	>3 months	2	5.30 [1.12, 9.47]	p = 0.01	0%	p<0.0001
****C-peptide****						
**** Type of surgery****						
	DJB	2	-0.11 [-0.38, 0.16]	p = 0.48	0%	p = 0.42
**** Duration of diabetes****						
	<8 years	2	-0.11[-0.38, 0.16]	p = 0.48	0%	p = 0.42
****Total cholesterol****						
**** Follow-up period****						
	>7 months	2	54.89[46.09,63.68]	p = 0.46	0%	p<0.00001
	<7 months	2	4.00 [2.45, 5.54]	p = 0.42	0%	p<0.00001
****Triglyceride****						
**** Follow-up period****						
	>7 months	2	15.66[12.68,18.64]	p = 0.98	0%	p<0.00001
**** Ethnics****						
	South America	2	89.43[19.92,198.79]	p = 0.10	64%	p = 0.11
****HOMA-IR****						
**** Follow-up period****						
	>3 months	2	4.46 [3.64, 5.28]	p = 0.61	0%	p<0.00001

BMI = body mass index; CI = confidence interval; FBG = fasting blood glucose; DJB = duodenojejunal bypass; HOMA-IR = homeostatic model of insulin resistance; PP = postprandial blood glucose

## Discussion

T2DM is a chronic disease with a high prevalence and limited major treatments. Bariatric surgery is suggested be an alternative treatment for T2DM that has possibilities to induce remission of the disease. In 2011, the International Diabetes Federation (IDF) released a statement [19] suggesting that bariatric surgery could be used in obese patients with a BMI >40 kg/m^2^ and that it might bring benefit to obese T2DM patients with a relatively low BMI (BMI 30~35 kg/m^2^), who do not respond to standard medical therapies. However, the effects of bariatric surgery for non-obese T2DM patients have not been established. Although previously published studies predominantly focused on the role of bariatric surgery in patients with BMI<35kg/m^2^, patients with BMI<30kg/m^2^ were occasionally included in most studies [20–22]. The global cutoff points of the BMI for overweight and obesity are set at 25.0 kg/m^2^ and 30.0 kg/m^2^, respectively, by the World Health Organization (WHO) [23]. However,epidemiological studies have suggested thatwhen the BMI exceeds 25kg/m^2^, every 5 kg/m^2^ elevation in the BMI is associated with an approximately 30% increase in mortality [24]. A systematic review of the co-morbidity incidences for overweight and obese populations in 89 studies showed that obese and overweight patients are associated with an increased risk of multiple co-morbidities [25]. Thus, overweight deserves attention equal to that of obesity in medical treatments, considering the incidence of morbidity and mortality. Additionally, several studies in overweight patients without obesity have suggested that surgery provided potential benefits in treating T2DM and its related co-morbidities [13,26] whereas some studies reported low remission and amelioration rates of only 15% and 30%, respectively [27]. Although our preliminary data suggested potential beneficial effects of surgery in non-obese T2DM patients, it is too early to suggest the clinical application of bariatric surgery for non-obese T2DM patients.

In this study, we pooled the data from 290 non-obese T2DM patients, who received bariatric surgery after the failure of glycemic control via the available pharmacological treatments. Statistically significant improvements in the HbA1c, FBG, PP and HOMA-IR levels aftersurgery were observed in the pooled analysis. Compared with the studies in patients with higher BMI levels, the trend was consistent; however, the level of improvement was lower. Our results suggested that various metabolic surgeries could lead to significant reduction in insulin administration, as well as the use of oral medication, regardless of the surgical procedure. Although no deaths were reported in the included studies, the overall major complication rate from surgery was 6.2%, and the reoperation rate was 1.7%, almost twice as much as the rates in patients with slightly higher BMI levels [20]. The ethical consideration should be drawn to seek approximately 40% remission rate of diabetes from surgery with a 6% major complication rate. Thus, the evidence remained far from adequate for supporting surgery in non-obese T2DM patients. Studies should report their data with prolonged follow-up periods to address the long-term effects and safety. The ethical considerations of newly conducted clinical trials should be analyzed carefully because the net benefit from surgical treatment in non-obese patients is difficult to address.

In our pooled results, a weight loss of 9.7 kg (ranging from 5.7% to 32%) and a remission rate of 42.4%(defined as the achievement of an HBA1c level < 6%) were observed after surgery. However, this finding was inconsistent with a meta-analysis conducted by Li et al., which involved 357 patients with a BMI <35 kg/m^2^ from thirteen studies, and reported a weight loss of 17.23kgwith an 80% remission rate(HBA1c<7%) and a 66.35%clinical resolution rate [20]. This inconsistency might suggest an association between the effect of bariatric surgery and the preoperative BMI. Patients with BMI 30~35 kg/m^2^ could increase the pooled effect size of surgery in the previous meta-analysis.

Our subgroup analysis indicated that patients with a longer follow-up duration might gain more benefit from the surgery, noting that the 95% CI was not overlapped, which indicated that a long-term reduction of the BMI and HbA1c levels derived by bariatric surgery in non-obese patients might be worth further investigation. The continuation of the follow-up of patients in existing studies is necessary to address the long-term effect and safety profiles. In the subgroup analysis, the patients with a longer duration of diabetes achieved a higher reduction of the BMI and HbA1c; however the 95% CI overlapped. This result is inconsistent with previous meta-analyses [16,28–34]. Traditionally, patients with a shorter diabetic duration were considered to have better beta-cell function. However, the patients with various durations of diabetes from our included studies had a similar pre-surgical C-peptide level, which is generally lower than that shown in most previous studies of bariatric surgery in patients with higher BMI levels [35,36]. In addition, our results indicated that the C-peptide level was not changed significantly after surgery, whereas only the HOMA-IR improved. The relatively poor beta-cell function in non-obese patients, regardless of the diabetic duration, might partially explain this inconsistency, as well as the poor efficacy of bariatric surgery in such patients. Only a few included articles reported the association of diabetes with its co-morbidities, adding difficulty in the accurate assessment of the metabolic status after surgery. With the available studies pooled, our overall results showed that TC and TG were not changed significantly after surgery. There remains no evidence for introducing bariatric surgery aiming to treat any co-morbidity in non-obese patients.

This meta-analysis has several limitations. First, our meta-analysis included a very limited number of non-RCT studies, which had small sample sizes and incomplete data, which potentially affected the accuracy of the analysis. Second, we used the BMI to define obesity and overweight, as suggested by the WHO, which could only partially reflect the fitness of diabetic patients. Third, significant heterogeneity was observed in the overall analysis, which might result in the pooled results being less convincing, although we applied random-effect models and conducted the subgroup analysis accordingly. Fourth, reporting bias could be introduced because positive results are more likely to be published. Fifth, only articles published in English were included, which might lead to publication bias. Finally, the follow-up durations of the included studies were short, which caused the long-term effect and safety data for non-obese patients to be unclear.

## Conclusion

Based on the currently available data, bariatric surgery might improve glycemic control and weight loss in a very limited range, with doubled surgical complications in drug-refractory T2DM patients with BMI <30 kg/m^2^. It is too early to suggest bariatric surgery for non-obese T2DM patients. Persistent follow-up of non-obese patients in existing studies is necessary to further address the long-term efficacy and safety of surgical treatment in this patient population.

## Supporting Information

S1 Table The preferred reporting items for systematic reviews and meta-analysis (PRISMA) checklist. (DOCX)

S2 Table Assessment of quality of the included studies for meta-analysis. (DOCX)

S3 Table Major side effects reported in the included studies. BAGUA = one anastomosis gastric bypass; BPD = biliopancreatic diversion; DJB = duodenojejunal bypass; LII-DSG = laparoscopic sleeve gastrectomy; LMGB = laparoscopic mini gastric bypass; RYGB = roux-en-Y gastric bypass.
(DOCX)

S4 Table Diabetes remission, clinical status of diabetes resolution, co-morbidity changes and safety of surgery in the included studies. LDL = low-density lipoprotein; NA = not available.
(DOCX)
