# Supplementary material for: Bariatric Surgery for Type 2 Diabetes Mellitus in Patients with BMI <30 kg/m2: A Systematic Review and Meta-Analysis
Source: PLoS One. 2015 Jul 13;10(7):e0132335. doi: 10.1371/journal.pone.0132335 (PMC4500506; doi:10.1371/journal.pone.0132335)
Supplement: S2 Table — (DOCX) [file pone.0132335.s002.docx]

**S2 Table. Assessment of quality of the included studies for meta-analysis**

|  | **Selection** | | **Comparability** | **Exposure/Outcome** | |
| --- | --- | --- | --- | --- | --- |
| **Studies** | **Were characteristics of subjects clearly described?** | **Were subjects representative of the entire population?** | **Was the study controlled for confounders adequate?** | **Was the ascertainment of exposure/ outcome clearly described?** | **Was the follow up long enough?** |
| Ramos et al | Yes | Yes | Yes | Yes | Yes |
| Depaula et al | Yes | Yes | Yes | Yes | Yes |
| Geloneze et al | Yes | Yes | Yes | Yes | Yes |
| Lee et al | Yes | No | Yes | No | Yes |
| Kim et al | Yes | Yes | Yes | Yes | Yes |
| Scopirano et al | Yes | Yes | Yes | Yes | Yes |
| Navarette et al | Yes | Yes | Yes | Yes | Yes |
| M. García et al | Yes | Yes | Yes | Yes | Yes |
| J.B. Dixon et al | Yes | Yes | Yes | No | Yes |
| C shrestha et al | Yes | Yes | Yes | Yes | No |
