# Supplementary material for: Bariatric Surgery for Type 2 Diabetes Mellitus in Patients with BMI <30 kg/m2: A Systematic Review and Meta-Analysis
Source: PLoS One. 2015 Jul 13;10(7):e0132335. doi: 10.1371/journal.pone.0132335 (PMC4500506; doi:10.1371/journal.pone.0132335)
Supplement: S3 Table — BAGUA = one anastomosis gastric bypass; BPD = biliopancreatic diversion; DJB = duodenojejunal bypass; LII-DSG = laparoscopic sleeve gastrectomy; LMGB = laparoscopic mini gastric bypass; RYGB = roux-en-Y gastric bypass. (DOCX) [file pone.0132335.s003.docx]

**S3 Table. Major side effects reported in the included studies**

| **Included study** | **Type of surgery** | **Side events** | | **Number of patients** |
| --- | --- | --- | --- | --- |
|  |  | **Early (<30 days)** | **Late (>30 days)** |  |
| Ramos et al | DJB | None | None | 0 |
| Depaula et al | LII+DSG | Fistula (n=1), gastrointestinal bleeding (n=1), urinary tract infection (n=1), pneumonia (n=2) | Prolonged diarrhea (n=2, 2.9%), gout attack (n=2, 2.9%), prolonged emesis (n=3, 4.4%), urinary tract  infection (n=3, 4.4%), and fungal esophagitis (n=1, 1.4%). Reoperation was done in 3 patients. | 5 |
| Geloneze et al | DJB | Minor hypoglycemic events (n=3) | None | 3 |
| Lee et al | DJB | Marginal ulcer and illeus (n=2). | None | 2 |
| Kim et al | LMGB | None | None | 0 |
| Navarette et al | LSG+DJB | Intraabdominal bleeding (n=1) | None | 1 |
| Scopirano et al | BPD | Intraperitoneal Bleeding (n=1) | Prolonged diarrhea (n=1)  Reoperation (n=1) | 1 |
| M. García et al | BAGUA | Not reported | Not reported | 0 |
| J.B. Dixon et al | MGB+RYGB | Gastrointestinal bleeding (n=2) | Anemia (n=34, 33%), Marginal ulcer (n=7, 7%), Reﬂux esophagitis  (n=10, 10%), Osteopenia (n=4, 4%) | 2 |
| C shrestha et al | - | Not reported | Not reported | 0 |
| Total | | | | 6.2% (n=14/225) |

BAGUA = one anastomosis gastric bypass; BPD = biliopancreatic diversion; DJB = duodenojejunal bypass; LII-DSG = laparoscopic sleeve gastrectomy; LMGB = laparoscopic mini gastric bypass; RYGB = roux-en-Y gastric bypass
