# Supplementary material for: Bariatric Surgery for Type 2 Diabetes Mellitus in Patients with BMI <30 kg/m2: A Systematic Review and Meta-Analysis
Source: PLoS One. 2015 Jul 13;10(7):e0132335. doi: 10.1371/journal.pone.0132335 (PMC4500506; doi:10.1371/journal.pone.0132335)
Supplement: S4 Table — LDL = low-density lipoprotein; NA = not available. (DOCX) [file pone.0132335.s004.docx]

**S4 Table. Diabetes remission, clinical status of diabetes resolution, co-morbidity changes and safety of surgery in the included studies**

| **Included studies** | **Insulin users before surgery**  **(%, n)** | **Diabetes related co-morbidities and complications** | | **Remission (%, n)** | | **Patients off medication (%, n)** | **Insulin users after surgery (%, n)** | **Safety of surgery** | |
| --- | --- | --- | --- | --- | --- | --- | --- | --- | --- |
|  |  |  |  |  |  |  |  | **Surgical complication rate (%)** | **Mortality** |
|  |  | **Status before surgery** | **Improvement rate after surgery** |  |  |  |  |  |  |
| Ramos et al | 0% (n=0) | - | - |  | - | 90% (n=18/20) | - | None | None |
| Depaula et al | 44% (n=30/69) | Dyslipedemia: 72.5% (n=50)  Hypertension: 66.7% (n=46)  Retinopathy: 26.1% (n=18)  Neuropathy: 24.6% (n=17)  Microalbuminuria: 80% (n=55)  Macroalbuminuria: 20% (n=14) | Hypertension controlled: 91.3 % (n=42)  Hypercholesterolemia normalized: 95% (n=47)  Hypertriglyceridemia normalized: 92% (n=46)  Retinopathy resolved: 44.4% (n=8)  Microalbuminuria resolved: 87.5% (n=48)  Macroalbuminuria was completely resolved.  Neuropathy resolved: 70.5% (n=12) |  | 65.2% (n=45/69) | 95.7% (n=66/69) | 0% (n=0/30) | 7.2% | None |
| Geloneze et al | 100% (n=12/12) | Dyslipedemia: 100% (n=12/12) | All patients’ value decreased than pre-operative period. |  | 16.7% (n=2/12) | None | 16.7% (n=2/12) | 25% | None |
| Lee et al | 33.34% (n=2/6) | - | - |  | - | None | 0% (n=0/2) | 33.3% | None |
| Kim et al | 40% (n=4/10) | - | - |  | 70% (n=7/10) | - | NA | None | None |
| Scopirano et al | 53.34% (n=8/15) | Hypertension: 33% (n=5/15)  Hypertriglyceridemia: 20% (n=3/15)  Hypercholesterolemia: 53% (n=8/15) | Hypertension controlled: 20% (n=3/15).  Hypertriglyceridemia normalized: 0% (0/15)  Hypercholesterolemia normalized: 100% (n=8/8) | 27% (n=4/15) | | 26.7% (n=4/15) | 37.5% (n=3/8) | 6.6% | None |
| Navarette et al | 100% (n=10/10) | Dyslipedemia: 90% (n=9/10) | All patients’ value was normalized in postoperative period. | 40% (n=4/10) | | 40% (n=4/10) | 0% (n=0/10) | 10% | None |
| M.García et al | 77% (n=10/13) | Dyslipedemia: 38.46% (n=5/13)  Hypertension: 92.3% (n=12/13)  Heart disease: 38.46% (n=5/13) | Serum level of cholesterol, triglyceride & LDL decreased significantly  Hypertension controlled: 100% (n=12/12)  Medication for heart disease decreased. | 77% (n=10/13) | | 23.1% (n=3/13) | 100% (n=10/10) | NA | None |
| J.B.Dixon et al | 31% (n=32/103) | - | - | 30.1% (n=31/103) | | - | NA | 1.9% | None |
| C.Shrestha et al | 24.2% (n=8/33) | Dyslipedemia: 100% (n=33/33) | All patients showed significant reduction in triglyceride.  All patients’ value regarding cholesterol decreased than baseline. | - | | 24.2% (n=8/33) | 50% (n=4/8) | - | None |
| Total | 42.8% (n=116/271) |  | | 44.3% (n=103/232) | | 64.3% (n=103/160) | 12.0% (n=19/158) | 6.2% | 0% |

LDL = low-density lipoprotein; NA = not available
